# Aggressive Clinical Course of Mucinous Tubular and Spindle Cell Renal Cell Carcinoma Following CT-Guided Ablation: A Case Report

**DOI:** 10.7759/cureus.94354

**Published:** 2025-10-11

**Authors:** Jonathan McAdam, Andrew McAdam, David Curry

**Affiliations:** 1 Urology, Belfast Health and Social Care Trust, Belfast, GBR

**Keywords:** ablation, mucinous tubular, renal cell carcinoma, spindle cell sarcoma, tumour thrombus

## Abstract

Mucinous tubular and spindle cell renal cell carcinoma (MTSRCC) is an uncommon renal epithelial neoplasm historically regarded as indolent. We describe an 81-year-old woman with an incidentally detected 1.4 cm right interpolar renal mass, biopsied and ablated percutaneously, with histology confirming World Health Organization/International Society of Urological Pathology grade 2 (WHO/ISUP grade 2) MTSRCC. Within three months, she developed extensive right renal vein thrombosis with propagation to the inferior vena cava (IVC) and right atrium, initially considered treatment-related. Despite systemic anticoagulation, she demonstrated rapid progression with innumerable hepatic metastases on magnetic resonance cholangiopancreatography (MRCP) six months after ablation and died shortly thereafter. This report underscores that MTSRCC can behave aggressively, with venous tumor thrombus and an explosive metastatic dissemination. It also highlights the diagnostic challenges distinguishing bland from malignant thrombus, as well as the nuances of managing small renal masses in frail older adults.

## Introduction

Mucinous tubular and spindle cell renal cell carcinoma (MTSRCC) is a rare renal cell carcinoma (RCC) subtype recognized in modern WHO classifications and characterized morphologically by tightly packed tubules merging with bland spindle cells in variably mucinous stroma. Although many series portray a low-grade, slow-growing phenotype, contemporary literature recognizes a spectrum of behaviour, including high-grade transformation, sarcomatoid change, nodal and visceral metastases, and more rarely, venous tumour thrombus. Recent case series and reviews since 2020 reiterate this heterogeneity and formalize MTSRCC as a distinct entity within the 2022 WHO framework [1-3]. Immunophenotypically, MTSRCC overlaps with papillary RCC (often cytokeratin 7 (CK7) and alpha methylacyl CoA racemase (AMACR) positive, carbonic anhydrase IX (CAIX) negative, variable cluster of differentiation (CD)10), but genomic studies show it is not merely a papillary variant. Copy-number analyses typically reveal multiple chromosomal losses (one, four, six, eight, nine, 13, 14, 15, 22) without classic papillary gains of chromosomes seven and 17, and aggressive cases have been associated with cyclin-dependent kinase inhibitor 2A/B (CDKN2A/B) loss and complex copy-number changes [4,5].

## Case presentation

**Figure 1:**
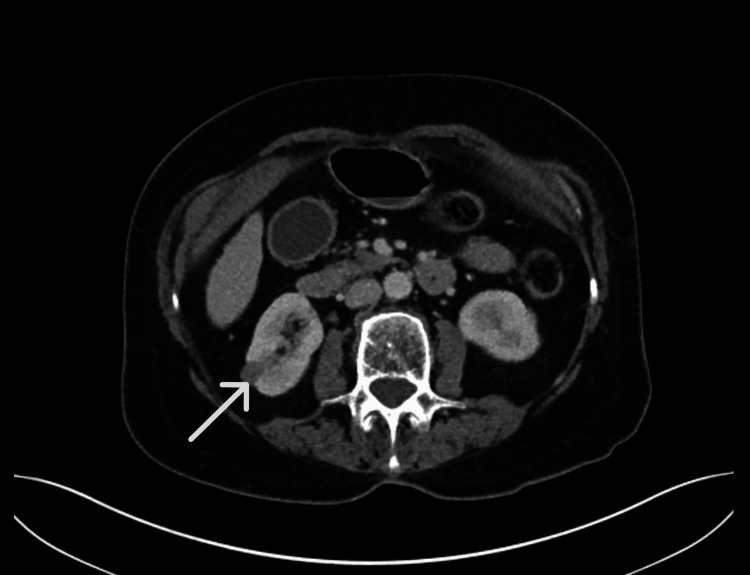
Axial CT renal Axial CT renal demonstrating a right-sided renal lesion (indicated by the arrow).

An 81-year-old woman was admitted with choledocholithiasis to the emergency department. She had a background of chronic kidney disease (CKD) stage 3, hiatus hernia, and sciatica. Her Eastern Cooperative Oncology Group (ECOG) performance status was 1. Contrast-enhanced CT during admission incidentally identified a 12 mm indeterminate lesion at the right lower pole (Figure 1). 

**Figure 2:**
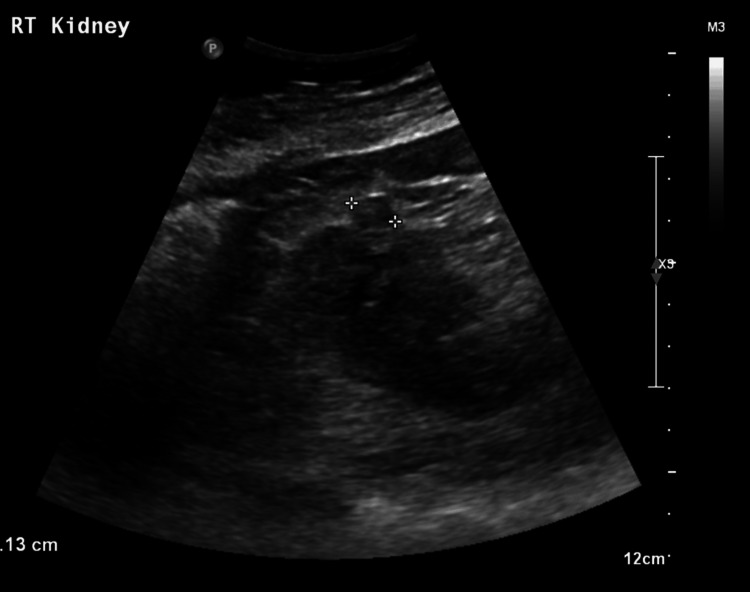
Ultrasound of the right kidney Ultrasound of the right kidney demonstrating the renal lesion.

An ultrasound, conducted the next day, showed a 1.1 cm (Figure 2) hyperechoic solid focus in the mid-lower pole with increased cortical echogenicity bilaterally. She remained asymptomatic without haematuria.

**Figure 3:**
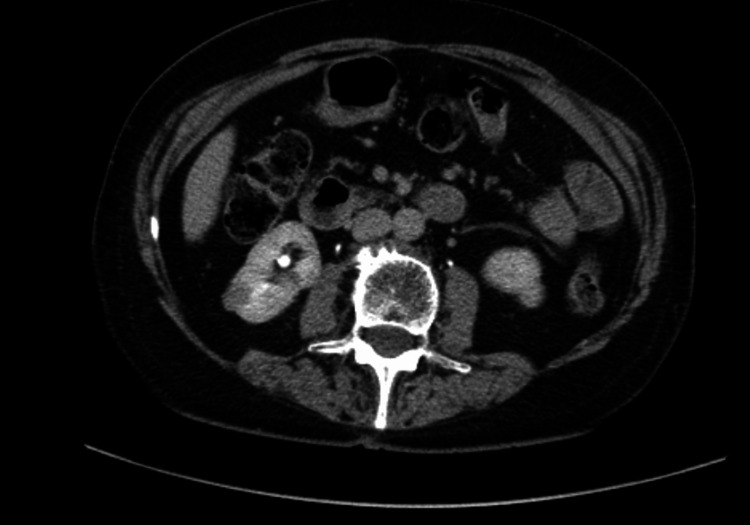
Interval axial CT abdomen 36 months after first imaging Interval axial CT demonstrating an increase in size of the renal lesion.

A CT colon study 36 months after the initial investigation, performed for an altered bowel habit, re-demonstrated a 1.3 cm right interpolar cystic lesion with internal septations (attenuation 65 HU). A dedicated renal protocol CT a month later confirmed a 1.4 cm enhancing cortical lesion (pre-contrast ~50 HU, post-contrast ~70 HU), raising suspicion for a small solid tumour; and a separate lower-pole simple cyst was also noted (Figure 3).

**Figure 4:**
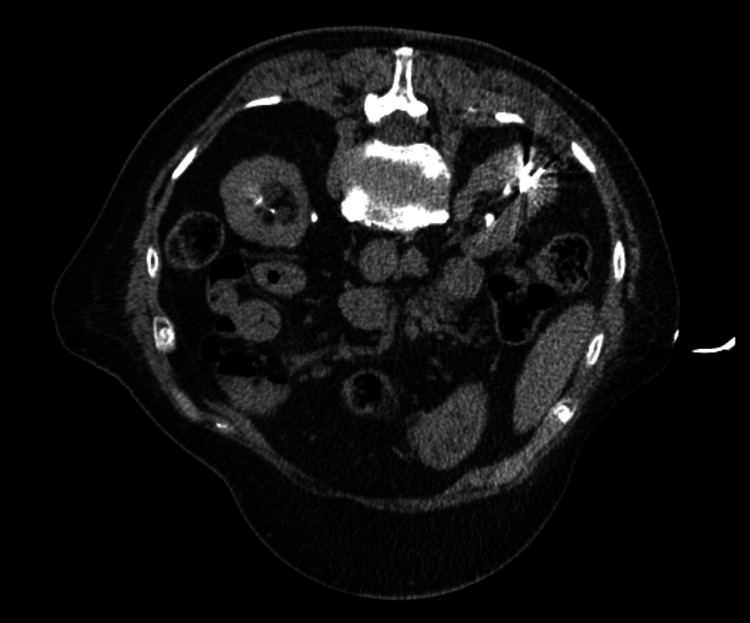
Axial CT demonstrating the ablation probes in the lesion during ablation

The case was discussed at the renal multidisciplinary meeting (MDM). In light of her pathology, comorbidities, and age, she was deemed appropriate for either active surveillance or ablation. Upon discussion with the patient, she was keen for ablation. Hence, five months after her renal CT, she proceeded to CT-guided core biopsy, with two cores being taken along with microwave ablation (Figure 4). 

Following ablation, a good treatment zone was achieved. 

**Figure 5:**
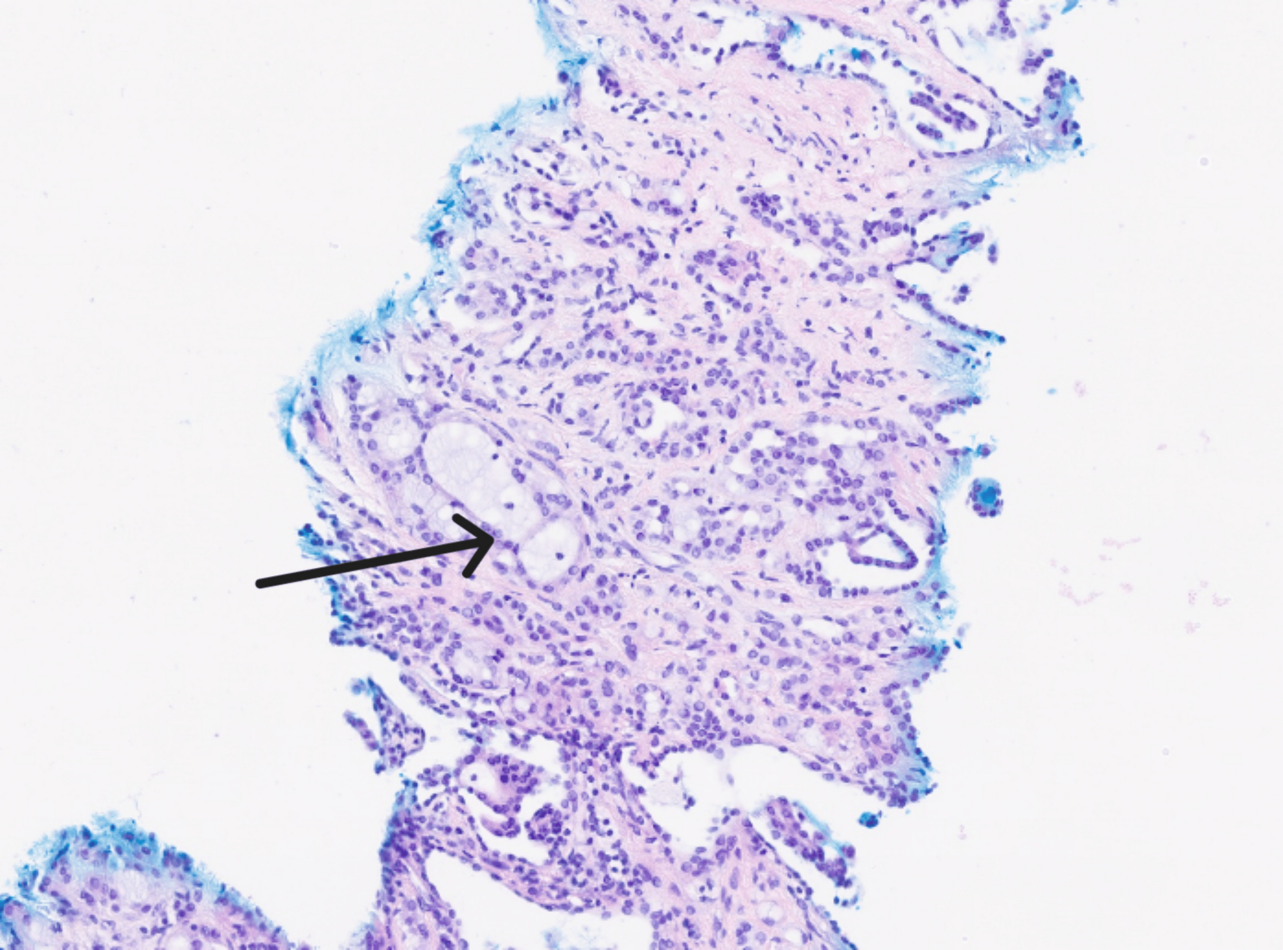
Histopathology specimen Histology specimen showing mucinous tubular and spindle cell carcinoma, World Health Organization/International Society of Urological Pathology grade 2 (WHO/ISUP grade 2).

Histology showed mucinous tubular and spindle cell carcinoma, World Health Organization/International Society of Urological Pathology grade 2 (WHO/ISUP grade 2), right kidney, unifocal cortical lesion; and two cores were obtained (longest 13 mm). Immunohistochemistry was positive for CK7, AMACR, Paired Box Gene 8 (PAX8), vimentin, epithelial membrane antigen (EMA), and succinate dehydrogenase subunit B (SDHB) and negative for CAIX, GATA binding protein 3 (GATA3), CD10, and Transcription Factor E3 (TFE3). No sarcoma/rhabdoid features, necrosis, or lympho-vascular invasion were identified (Figure 5).

**Figure 6:**
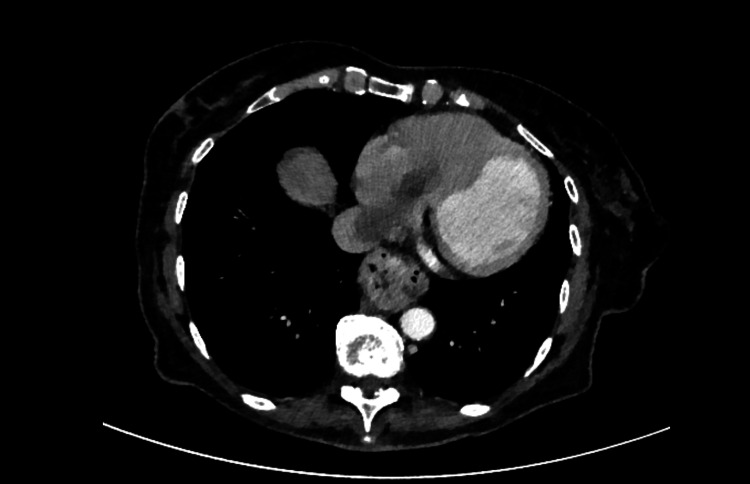
Axial CT chest with contrast CT chest which demonstrates the thrombus extending into the right atrium.

She initially recovered well and the MDM follow-up plan was for cross sectional imaging at three, 12, 24, and 36 months followed by alternate years for a total follow up period of 10 years. At each follow-up appointment she was to have her full blood picture, renal function, liver function and calcium checked. However, three months following the ablation she presented with right flank pain, microscopic haematuria, pyrexia, and was found on CT to have right renal parenchymal swelling and a 3 cm posterior cortical low-density area at the mid-pole, with extensive right renal vein thrombosis propagating cranially into the inferior vena cava (IVC) and right atrium. During admission under urology, therapeutic anticoagulation was commenced (clexane followed by warfarin). Vascular and cardiac input favoured a treatment-related thrombus rather than tumour thrombus at that time, and a repeat CT was planned (Figure 6).

**Figure 7:**
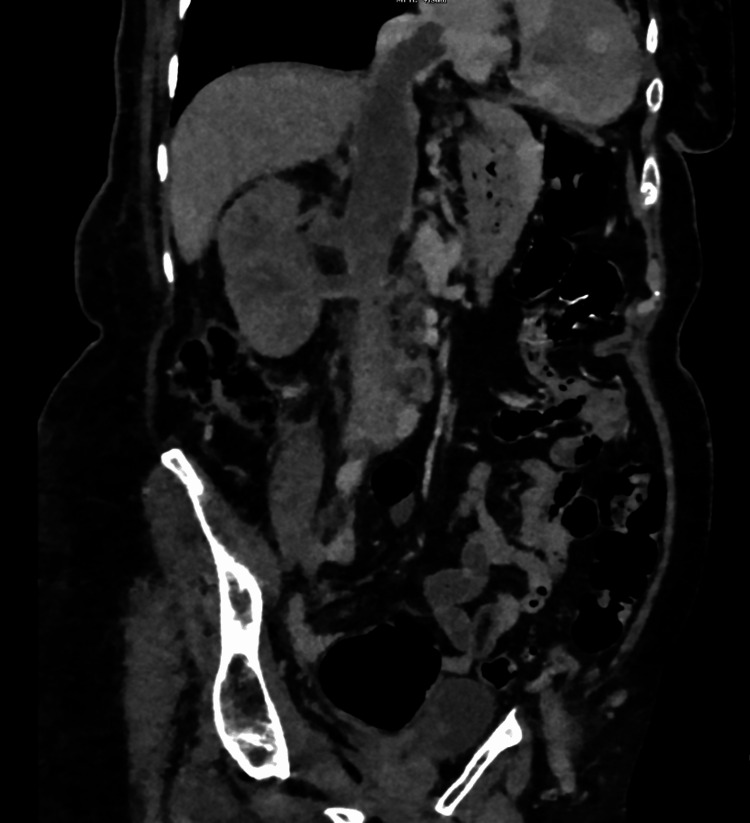
Coronal CT abdomen and pelvis CT demonstrating tumour thrombus extending up the inferior vena cava.

**Figure 8:**
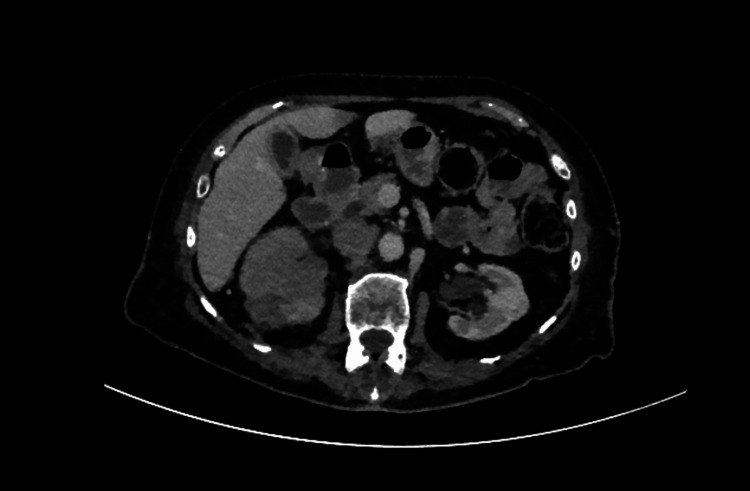
CT demonstrating indeterminate renal findings

She was readmitted with a lower respiratory tract infection the following month, when a CT again showed persistent renal vein/IVC/right-atrial thrombus, ill-defined right renal changes, and adjacent lymphadenopathy, raising suspicion of underlying malignancy (Figures 7, 8)

Transthoracic echocardiography demonstrated a large echodense mass prolapsing through the tricuspid valve from the IVC into the right atrium, consistent with thrombus. She was discharged on anticoagulation.

**Figure 9:**
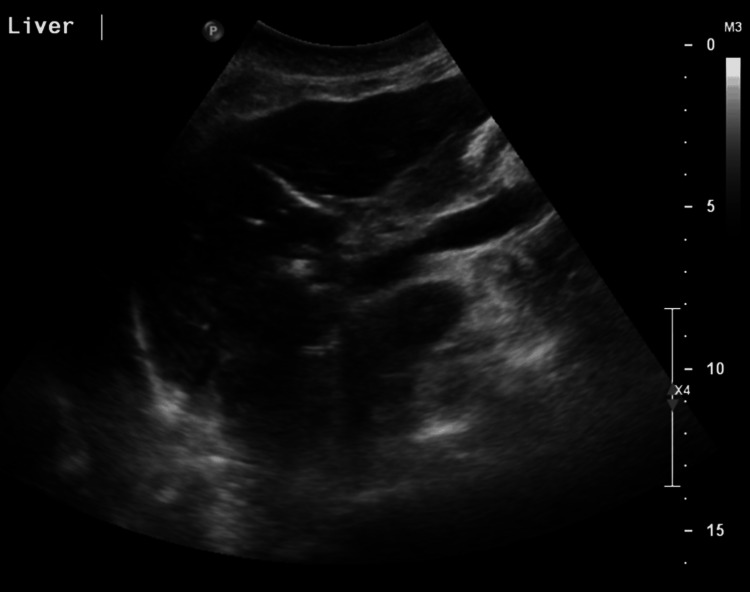
Ultrasound of the liver Ultrasound of the liver demonstrating the dilated external bile duct.

A Computed Tomography Chest, Abdomen, and Pelvis (CT-CAP) two weeks after discharge reported stable right renal findings but persistent pedunculated right-atrial thrombus and an indeterminate small right-upper-lobe pulmonary nodule. She required a further admission for symptomatic anaemia and hyponatraemia, was transfused, and remained stable when reviewed in the clinic following discharge. Four weeks following review in the clinic she presented again with shortness of breath and lethargy; she was anaemic (Hb 74 g/L; normal range: 120-155 g/L) with a coagulopathy (international normalized ratio or INR >10; normal range: 0.8-1.1), requiring reversal and transfusion, followed by ongoing clinical decline. Ultrasound showed extrahepatic biliary dilatation (common bile duct 11 mm; normal <7mm) without intrahepatic duct dilatation (Figure 9). 

**Figure 10:**
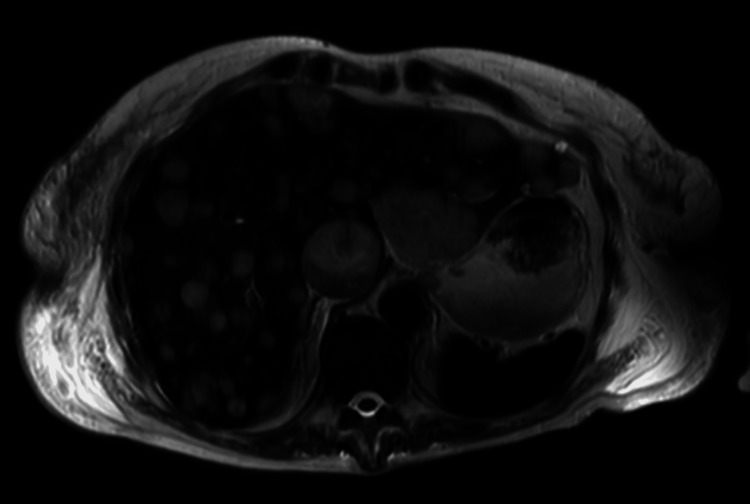
MRI of the liver MRI liver demonstrating multiple liver metastases.

Magnetic resonance cholangiopancreatography (MRCP) revealed massive hepatic infiltration by innumerable metastases, including a dominant left-lobe lesion not evident on CT seven weeks earlier (Figure 10).

**Figure 11:**
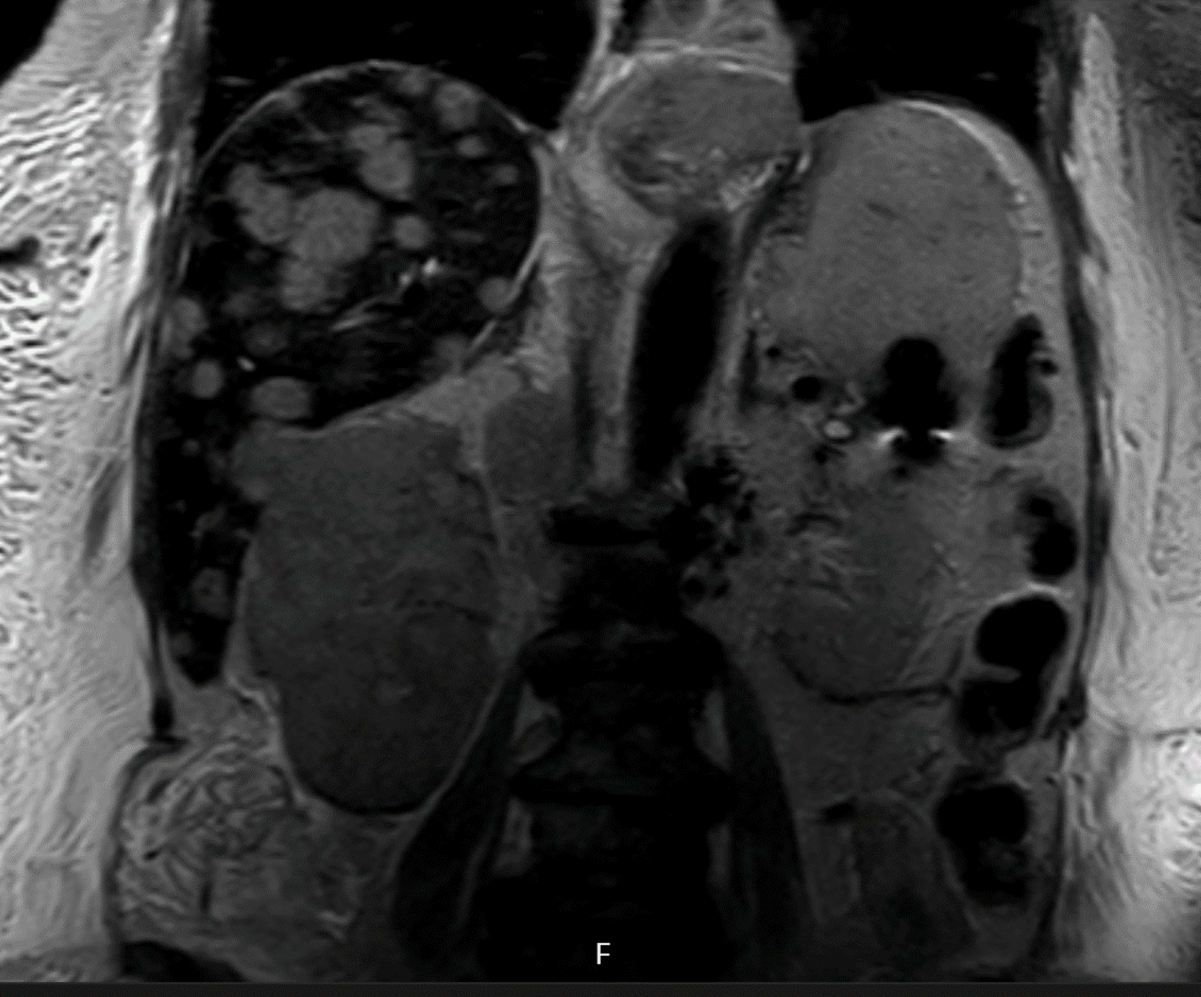
Coronal magnetic resonance cholangiopancreatography (MRCP) demonstrating diffuse right renal tumour

This was along with a diffuse right renal tumour (Figure 11) extending through the renal vein and IVC to the right atrium with prolapse through the tricuspid valve.

She was transitioned to comfort care and passed away nine days after her admission.

## Discussion

This case illustrates a fulminant course of MTSRCC, a tumour generally viewed as low-grade. Contemporary series emphasize that although many MTSRCCs are localised at diagnosis, a subset shows locally advanced disease, venous tumour thrombus, and metastatic spread. In a 2023 institutional series of seven MTSRCCs, one patient presented with a level-I IVC thrombus and died of disease at 24.5 months, underscoring that venous involvement can occur and portends worse outcomes [6,7]. Additional case reports describe high-grade MTSRCC with widespread metastases, including liver, and clinically aggressive deterioration [8]. The pathogenesis of the aggressive behaviour in MTSRCC remains incompletely defined. Molecular studies demonstrate that MTSRCC is distinct from papillary RCC, with characteristic broad chromosomal losses rather than the 7/17 gains typical of papillary RCC. Aggressive MTSRCCs have been linked to CDKN2A/B homozygous deletion and complex copy-number alterations, suggesting clonal evolution from an initially low-grade lesion [4,5]. These genomic insights mirror clinical heterogeneity and caution against assuming uniformly indolent behaviour based solely on histotype. A central challenge in this case was discriminating the bland thrombus from tumour thrombus after ablation. Cross-sectional imaging features favouring tumour thrombus include unequivocal enhancement within the thrombus, contiguity with a renal mass, vessel expansion, restricted diffusion on MRI, and 18-Fluoro-2-deoxy-D-glucose (FDG) avidity on Positron Emission Tomography (PET). In contrast, a bland thrombus shows no enhancement and may not expand the vessel. MRI offers high diagnostic accuracy for characterising venous thrombus and assessing caval wall invasion, and PET/CT can help differentiate tumour from bland thrombus when enhancement is equivocal [9-11]. In retrospect, earlier oncologic re-staging with contrast-enhanced MRI and/or PET might have clarified the nature of the thrombus sooner in this patient. Management decisions for small renal masses (SRMs) in frail older adults are complex. Contemporary guidelines from European and American societies endorse active surveillance or thermal ablation as acceptable options for cT1a lesions in patients with substantial comorbidity or limited life expectancy, acknowledging that cancer-specific mortality often remains low relative to competing risks in this population. There is, however, a preference for active surveillance in those over the age of 75 with significant comorbidities [12-15]. The chosen plan in this case (biopsy followed by ablation) fits within these consensus frameworks when factoring in patient preference and the relatively good functional baseline of the patient. Yet the subsequent course illustrates that even biopsy-proven low-grade histology does not preclude rapid progression. Whether the extensive venous thrombosis here was procedure-related or tumour-related cannot be proven. Post-ablation venous thrombotic events are uncommon but reported. Conversely, RCC with venous tumour thrombus, regardless of the histologic subtype, occurs in up to roughly 10% of cases and portends increased surgical complexity and recurrence risk [16,17]. The sheer extent of thrombus in this patient, in hindsight combined with subsequent explosive hepatic metastases, would be more suggestive of tumour thrombus rather than treatment-related. Two additional learning points emerged. First, MTSRCC may progress rapidly even from small primaries [8], reinforcing the value of dynamic risk assessment that integrates histology, interval events (e.g., thrombus), and patient frailty rather than relying on size and grade alone. Second, diagnostic uncertainty about thrombus biology should lower the threshold for advanced imaging and early repeat staging; enhancement-based criteria, diffusion characteristics, and metabolic imaging can guide escalation to oncologic management when a malignant thrombus is suspected.

## Conclusions

Although classically indolent, MTSRCC can display aggressive biology with venous tumour thrombus and rapid metastatic spread. In older, comorbid patients with small renal masses, both active surveillance and ablation are guideline-concordant. However, the appearance of venous thrombosis after focal therapy warrants urgent re-characterisation with MRI (and, when helpful, PET/CT) to distinguish bland thrombus from tumour thrombus and to restage comprehensively. This case adds to the growing literature that MTSRCC is heterogeneous and that vigilance is required to identify outlier biology early.
